# A new method for dealing with collider bias in the PWP model for recurrent events in randomized controlled trials

**DOI:** 10.1186/s12874-025-02596-0

**Published:** 2025-05-26

**Authors:** Chen Shi, Jia-Wei Wei, Zi-Shu Zhan, Xiao-Han Xu, Ze-Lin Yan, Chun-Quan Ou

**Affiliations:** 1 State Key Laboratory of Multi-organ Injury Prevention and Treatment, Department of Biostatistics, Guangdong Provincial Key Laboratory of Tropical Disease Research, School of Public Health, Southern Medical University, Guangzhou, 510515 China; 2 Novartis Institutes for Biomedical Research Co., Shanghai, China; 3 School of Biomedical Engineering, School of Information and Communication Engineering, Hainan University, Haikou, 570228 China; 4 Hainan Institute of Real World Data, Qionghai, 571437 China

**Keywords:** Recurrent events, Randomized controlled trials, Collider bias, PWP model, Treatment effects

## Abstract

**Background:**

Evaluating recurrent events within a time-to-event analysis framework effectively utilizes all relevant information to address the clinical question of interest fully and has certain advantages in randomized controlled trials (RCTs). However, the Prentice, Williams, and Peterson (PWP) model disrupts the randomness of the risk set for subsequent recurrent events other than the first and consequently introduces bias in estimating effects. This study aimed to propose a weighted PWP model, evaluate its statistical performance, and assess the potential consequences of using common practices when each recurrence has different baseline hazard functions.

**Methods:**

We proposed adjusting the estimate of treatment effect through a weighting strategy that constructed a virtual population balanced between groups in each risk set. A simulation study was carried out. The characteristic of the simulation data was the baseline hazard changed with the number of events. The proposed weighted PWP model was compared with current methods, including Cox for time-to-first-event, Poisson, negative binomial (NB), Andersen-Gill (AG), Lin-Wei-Yang-Ying (LWYY), and PWP models. Model performance was evaluated by bias, type I error rates, and statistical power. All models were applied to a real case from a randomization trial of Chemoprophylaxis treatment for Recurrent Stage I Bladder Tumors.

**Results:**

The results showed that the proposed weighted PWP model performed best with the lowest bias and highest statistical power. However, other models, including the Cox for time-to-first-event, Poisson, NB, AG, LWYY, and PWP models, all showed different degrees of bias and inflated type I error rates or low statistical power in the case of the baseline hazard changed with the number of events. Covariate adjustment via outcome regression can lead to inflated type I error rates. When the number of recurrent events was restricted, all weighting strategies yielded stable and nearly consistent results.

**Conclusions:**

Recurrent event data should be analyzed with caution. The proposed methods may be generalized to model recurrent events. Our findings serve as an important clarification of how to deal with collider bias in the PWP model in RCTs.

**Supplementary Information:**

The online version contains supplementary material available at 10.1186/s12874-025-02596-0.

## Introduction

In a longitudinal clinical study, each patient may experience clinical events of interest recurrently at various time points during the follow-up period. Examples of recurrent events include hospital admissions, migraines, cancer recurrences, and upper respiratory infections [1]. Such multiple event-time observations offer a temporal profile of the disease burden or progression in patients, providing valuable insights into their conditions [2]. An important consideration is the collective utilization of these observations, particularly when evaluating a new therapy versus standard care. Recurrent events are increasingly being considered as primary or secondary outcomes in randomized controlled trials (RCTs) [3, 4].

A common approach to analyzing recurrent events is through the recurrence rate based on the Poisson or Negative binomial (NB) model [5–8], which measures the average number of recurrences per unit of time. However, this approach relies on the strong assumption that the incidence of events remains constant over time [9]. Furthermore, even if the assumption holds, there may still be variations in the time it takes for each event to occur [10]. Therefore, evaluating recurrent events within a time-to-event analysis framework can be more informative. Standard survival analysis procedures focus on the time to the first event, analyzing the duration from enrollment or randomization to a specific event or the first occurrence of one of a set of pre-specified clinical events [11]. These approaches do not fully utilize all relevant information from all events, potentially limiting their ability to comprehensively address the clinical question of interest. Against this background, the extension of the Cox proportional hazard model to recurrent events has been actively pursued, primarily for evaluating time-to-recurrent events [12, 13].

Extensions of the original Cox model for recurrent event data mainly included the Andersen-Gill (AG) [12] and Prentice, Williams, and Peterson (PWP) [13] models. The AG model assumes a common baseline hazard function for all events, which may not align with the actual situation. For example, event dependency has been observed in cases such as falls [14] and hospitalizations in heart failure [15], where the baseline hazard significantly increases with the number of prior episodes. The PWP model analyzes ordered multiple events by stratification, assigning a specific baseline hazard function to each recurrence. The concept behind the PWP model is both reasonable and comprehensible. Nevertheless, the PWP model restricts the risk set for each event to individuals who have experienced the preceding event, probably introducing collider bias in estimating effects by disrupting the randomness of the risk set for subsequent recurrent events other than the first event in RCTs [16]. This disadvantage limits the use of the PWP model, although it has been employed in some observational studies and RCTs [17, 18]. The magnitude and characteristics of this bias, as well as methods for correcting it, require further study. The literature lacks a comprehensive evaluation of existing statistical methods for recurrent events when the baseline hazard function varies for each recurrence in RCTs.

To address these issues, in this study, we extended the PWP model by incorporating a weighting strategy to better estimate treatment effects in RCTs. In particular, we applied an entropy balance weighting framework [19] to reconstruct the randomness of the risk set for each recurrent event. Then, we performed Monte Carlo simulations to evaluate the statistical performance of the proposed method and assess the potential consequences of existing statistical methods in various settings. Finally, we demonstrated this method by applying it to the Bladder Cancer Recurrences data [20–22].

## Methods

### Sources of bias in the PWP model

The PWP models can be formulated in two distinct ways to summarize the treatment effect on recurrent events for RCTs, depending on the risk interval utilized [23]. Equation (1) and Eq. (2) are the hazard functions of PWP counting process (PWP-CP) and PWP gap time (PWP-GT), respectively.

1 $${h}_{ik}\left(t\right)={h}_{0k}\left(t\right){e}^{{A}_{i}\beta }$$

2 $${h}_{ik}\left(t\right)={h}_{0k}\left(t-{t}_{k-1}\right){e}^{{A}_{i}\beta }$$

where each individual $$i$$ ($$i=1,\dots ,n$$) is assigned to either the experimental treatment group ($${A}_{i}=1$$) or the control group ($${A}_{i}=0)$$. $$\beta$$ represents the corresponding coefficient, $$k$$ denotes the $$k$$th event for individual $$i$$, and $${h}_{0k}\left(t\right)$$ is the baseline hazard function at time $$t$$, which depends on $$k$$. $${t}_{k-1}$$ indicates the time at which the $$(k-1)$$th event occurs. We define the treatment effect as average cumulative hazard ratio $$\frac{{H}_{A=1}\left(t\right)}{{H}_{A=0}\left(t\right)}$$, a population-level causal estimand that compares the two types of potential outcomes up to $$t$$ over the superpopulation [24]. For any time $$t$$ and any weight $${\varphi }_{k}$$ ($$\sum_{k=1}^{K}{\varphi }_{k}=1$$), under the assumptions that (1) there is no interference between individuals, and there are no multiple versions of each treatment value leading to a different outcome (Stable Unit Treatment Value Assumption); (2) censoring is non-informative; (3) the proportional hazards assumption holds; (4) individuals have been randomized to treatment groups for each $$k$$ [25], we can estimate the above treatment effect as $$\frac{{H}_{A=1}\left(t\right)}{{H}_{A=0}\left(t\right)}=\frac{\sum_{k=1}^{K}{\varphi }_{k}{H}_{A=1,k}\left(t\right)}{\sum_{k=1}^{K}{\varphi }_{k}{H}_{A=0,k}\left(t\right)}=\frac{\sum_{k=1}^{K}{\varphi }_{k}{\int }_{0}^{t}{h}_{A=1,k}\left(u\right)du}{\sum_{k=1}^{K}{\varphi }_{k}{\int }_{0}^{t}{h}_{A=0,k}\left(u\right)du}=\frac{\sum_{k=1}^{K}{\varphi }_{k}{\int }_{0}^{t}{h}_{0k}\left(u\right){e}^{\beta }du}{\sum_{k=1}^{K}{\varphi }_{k}{\int }_{0}^{t}{h}_{0k}\left(u\right)du}={e}^{\beta }$$. Detailed proof is given in Method S1 (Supplementary Material 1). The above estimate is numerically equal to the value of one of the most commonly used summary measures in clinical trials: ratio of instantaneous hazard, i.e. hazard ratio (HR) [26]. However, the estimates of the traditional PWP model do not satisfy the fourth assumption and the explanations are as follows.

**Fig. 1:**
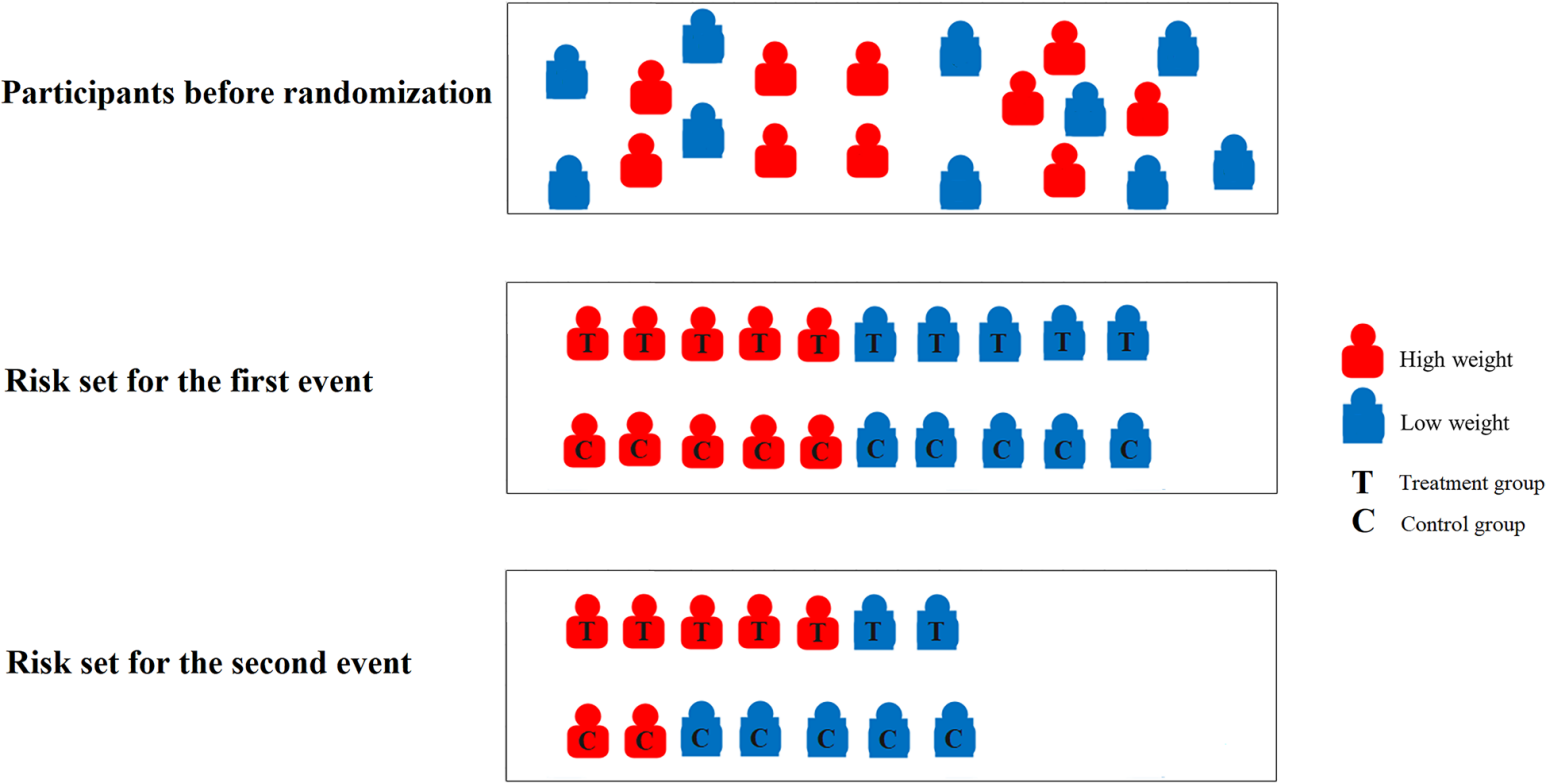
Schematic diagram of the risk set for PWP model

The $k$th risk set contains individuals at risk for the $k$th event. The PWP model assumes that the baseline hazard of an event varies depending on the number of previous events, with stratification by the number of events [27]. This approach includes all individuals in the first strata but only those who have experienced $k-1$ events in the *k*th event risk set. As illustrated in the schematic diagram of an RCT (Fig. 1), participants were randomly assigned to the control and treatment groups in a 1:1 ratio. Consider an extreme example in which the outcome event is only associated with two unrelated factors (treatment: protective effect; high weight: harmful effect). In this scenario, participants in the treatment group who experienced the first outcome event tended to be more high-weight than those in the control group. Consequently, when estimating the treatment effect using the PWP model, there will be an imbalance in weight in the second-event risk set between the two groups. As a result, even after randomization, biased effect estimates may still be obtained.

**Fig. 2:**
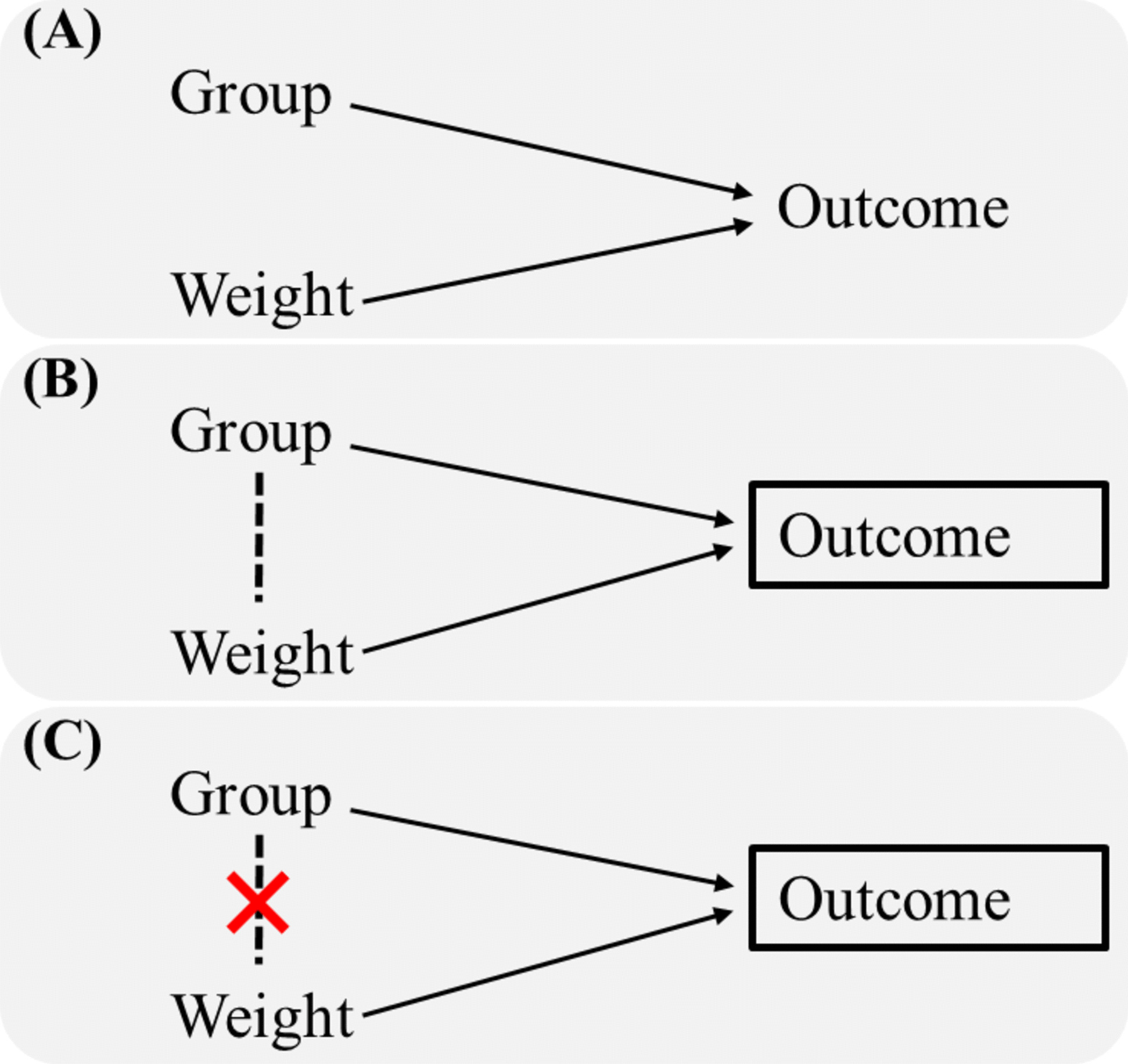
Directed acyclic graphs. The arrows represent hypothetical causal relationships. The rectangle indicates that the outcome is controlled for by analysis since the $(k+1)$th stratum analysis only includes patients who had experienced $k$th events. The dashed line represents a spurious association that may bias the estimation of the true group-outcome effect. The red cross indicates that the following weighting strategy eliminates the spurious associations

The weight does not influence the effect of treatment group on the outcome event after randomization (Fig. 2A). However, if the analysis is restricted to patients who had the outcome event, as done by limiting the risk set to individuals with prior events in the PWP model, a spurious association between group and weight is created (Fig. 2B). Specifically, when the $(k+1)$th stratum analysis only includes patients who had experienced $k$th events, which means the outcome (collider) is controlled for by analysis. Participants in the treatment group who experienced the outcome event tended to be more high-weight than those in the control group according to Fig. 1. Consequently, group and weight gradually become correlated with more high-weight individuals in the treatment group (Fig. 2B). This inadvertently introduces collider bias when estimating treatment effects using the PWP model (Eq. 1 or Eq. 2) [28], due to the confounding of treatment effect by weight.

### A new method proposed: PWP model with a weighting strategy

To solve the problem of collider bias in the PWP model, we proposed adjusting the estimate of treatment effect through a weighting strategy that constructed a virtual population balanced between groups in each risk set (Fig. 2C). Taking the PWP-GT model (hereafter, PWP model) as an example, the likelihood function is as follows:

3 $$L\left(\beta \right)=\prod_{i=1}^{N}\prod_{k=1}^{K}{(\frac{{e}^{{A}_{i}\beta }}{\sum_{j=1}^{N}{Y}_{kj}({t}_{ki}){e}^{{A}_{j}\beta }})}^{{\delta }_{ki}} , {Y}_{ik}\left(t\right)=I({t}_{ki}-{t}_{k-1,i}>t)$$

Here, $N$ is the sample size, $K$ is the maximum number of recurrent events, ${\delta }_{ki}$ is the censoring variable indicating whether the $k$th event is observed (${\delta }_{ki}=1$) or censored (${\delta }_{ki}=0$) for the $i$th individual.

The weight for individual $i$ in the $k$th risk set is denoted as ${\omega }_{ki}$. We considered using weights obtained by entropy balance to construct a balanced virtual population. Entropy balance has been demonstrated to be more efficient than the iterative propensity score approach and can ensure covariate balance within a pre-specified tolerance [29]. Additionally, entropy balance is doubly robust in that it addresses concerns about the bias in treatment effect estimation due to model misspecification [30].

In general, let $A$ denote the binary treatment indicator ($A=0$ for placebo, $A=1$ for experimental treatment). For individuals in the first risk set, since they maintain the initial randomization, the weight vector ${\omega }_{1}=(1,\dots ,1)$. For the ${N}_{k}$ individuals in the $k$th risk set ($k>1$), the weight vector ${\omega }_{k}=({\omega }_{k1},\dots ,{\omega }_{k{N}_{k}})$ is obtained by minimizing the relative entropy $D({\omega }_{k}\parallel {d}_{k})$, between ${\omega }_{k}$ and a vector of reference weights ${d}_{k}=({d}_{k1},\dots ,{d}_{k{N}_{k}})$ while imposing empirical mean balance with respect to the functions ${u}_{k1}({X}_{cov}),\dots ,{u}_{kP}({X}_{cov})$. To be specific, ${\omega }_{k}$ is found by solving the optimization problem:

4 $$\underset{{\omega }_{k}}{\text{min}}D({\omega }_{k}\parallel {d}_{k})$$

subject to the balance and normalizing constraints:

5 $$\begin{array}{c} \frac{1}{{N}_{k,A=1}}\sum\limits_{\left\{i:{A}_{i}=1\right\}}{\omega }_{ki}{u}_{kp}\left({X}_{cov, i}\right)\\ =\frac{1}{{N}_{k,A=0}}\sum\limits_{\left\{i:{A}_{i}=0\right\}}{\omega }_{ki}{u}_{kp}\left({X}_{cov,i}\right) =\frac{1}{{N}_{k}}\sum_{i=1}^{{N}_{k}}{u}_{kp}({X}_{cov,i})={\widehat{m}}_{kp} \end{array}$$

Where $p=1,\dots , P$, ${m}_{kp}=E({u}_{kp}({X}_{cov,i}))$ and ${\widehat{m}}_{kp}$ is the estimator of ${m}_{kp}$. $\sum_{\{i:{A}_{i}=1\}}{\omega }_{ki}={N}_{k, A=1}$ and $\sum_{\{i:{A}_{i}=0\}}{\omega }_{ki}={N}_{k, A=0}$ represent the number of individuals in the experimental treatment group and control group, respectively, in the $k$th risk set.

There are two important points involved in the above weighting process. First, the relative entropy $D({\omega }_{k}\parallel {d}_{k})$ in Eq. (4) measures the divergence between the distribution of estimated weights ${\omega }_{k}$ and the distribution of the reference weights ${d}_{k}$. The reference weights are considered as fixed and can represent some prior information. We use the set of weights with ${d}_{k}=(\frac{1}{{N}_{k}},\dots ,\frac{1}{{N}_{k}})$ as our reference weights to ensure that the weights are as uniform as possible and avoid extreme values [19]. The relative entropy $D({\omega }_{k}\parallel {d}_{k})$ is a divergence metric chosen from a member of the *Rényi divergences*, defined by: $D\left({\omega }_{k}\parallel {d}_{k}\right)={D}_{\alpha }\left({\omega }_{k}\parallel {d}_{k}\right)=\frac{1}{\alpha -1}\text{log}(\sum_{i=1}^{{N}_{k}}\frac{{\omega }_{ki}^{\alpha }}{{d}_{ki}^{\alpha -1}})$, $\alpha>0$. We chose the most used *Rényi divergences*: the Kullback–Leibler divergence (KL) ($\alpha =1$), given by ${D}_{1}\left({\omega }_{k}\parallel {d}_{k}\right)={\sum }_{i=1}^{{N}_{k}}{\omega }_{ki}\text{log}\frac{{\omega }_{ki}}{{d}_{ki}}$ to measure the divergence between the distribution of ${\omega }_{k}$ and ${d}_{k}$ [31]. Second, the balance constraints are defined in Eq. (5). ${X}_{cov}$ denotes $P$ dimension additional influencing factors that need to be considered at randomization, and ${u}_{kp}({X}_{cov})$ is the real-valued function for the $p$th dimension of ${X}_{cov}$. The function ${u}_{kp}(\bullet )$ means moment functions of the covariates. They are implemented to equalize the moments of the covariate distributions between the weighted treatment group and the control group. We chose the first order moment as a constraint for determining the weights. A previous study [31] has detailed how to optimize $\underset{{\omega }_{k}}{\text{min}}D({\omega }_{k}\parallel {d}_{k})$ and obtain entropy balance estimators.

To evaluate the treatment effect using the weighted PWP model, we constructed the likelihood function. According to Eq. (3), the stratified weighted partial likelihood score function equals the summation of $K$ event-specific functions, yielding the score equation for $\beta$:

$$\sum_{k=1}^{K}\left\{\sum_{i=1}^{N}{\omega }_{ki}{{\delta }_{ki}A}_{i}-\sum_{i=1}^{N}{\omega }_{ki}{\delta }_{ki}\frac{\sum_{j=1}^{N}{Y}_{kj}\left({t}_{ki}\right){\omega }_{kj}{A}_{j}{e}^{{A}_{j}\beta }}{\sum_{j=1}^{N}{Y}_{kj}\left({t}_{ki}\right){\omega }_{kj}{e}^{{A}_{j}\beta }}\right\}=0$$

where ${\omega }_{ki}=1$, if $k=1$; otherwise, ${\omega }_{ki}$ is obtained by entropy balance if $k>1$. Additionally, we used the robust (sandwich) estimator [32, 33] of the variance with the individual as the cluster to account for dependence between events within individuals. In practice, the data may need to be limited to a specific number of recurrent events if the risk set becomes very small for later strata. We restricted the number of recurrent events to those that included all treatment groups and at least two individuals in each group, ensuring that entropy balance estimators could function properly.

## Simulation study

This simulation study was conducted and reported using “Aims, Data generating process, Method of analysis, Estimands and Performance” approach [34], providing a scientifically coherent structured framework for designing, interpreting, and reporting simulation studies. All simulations and analyses were conducted using R 4.1.3 (R Foundation for Statistical Computing).

### Aims

The primary aim of this simulation study was to evaluate whether using entropy balance weights improves the estimation of the PWP model in the presence of collider bias under various covariate effects and sample sizes. The performance of the proposed weighted PWP model was compared against common methods for handling recurrent events. Additionally, we examined the impact of choosing inverse probability weighting instead of entropy balance weights on effect estimation [35]. Lastly, we assessed the effects of a truncated strategy on imbalanced event strata after weighting.

### Data generating mechanisms

The data generating mechanism in this simulation study focused on specifying different baseline hazard functions for each recurrent event. We simulated data using Weibull distributions with different parameters within each recurrence [36]. The survival function for each recurrent event is denoted as $S\left(t\right)={e}^{-\lambda {t}^{q}}$ and the density function as $f\left(t\right)=\lambda p{t}^{q-1}{e}^{\left(-\lambda {t}^{q}\right)}$, where $\lambda ={e}^{-q({\beta }_{0}+A\beta +{X}_{cov}\gamma )}$.

The combination of $q$ and ${\beta }_{0}$ defines the baseline hazard function. Influencing factors include treatment and other covariates, with the treatment effect size represented as $\text{HR}={e}^{-q\beta }$ and additional covariate effect represented as ${\text{HR}}_{cov}={e}^{-q{\gamma }_{p}}$. Observation time ${t}_{k}$ was simulated only for subjects with an event in ${t}_{k-1}$.

The simulation parameters of four scenarios are summarized in Table 1. The maximum number of recurrent events that a subject could experience was not fixed, but the baseline hazard was set to be constant when $k\ge 5$. Influencing factors were represented by a treatment indicator $T\sim Bernoulli (0.5)$ and five independent covariates with ${\text{HR}}_{cov}=0.9$ or ${\text{HR}}_{cov}=1.2$. The treatment effect size was set as $\text{HR}=0.75$ to assess bias and statistical power and $\text{HR}=1$ to evaluate type I error (T1E) rates for rejecting the null hypothesis of no treatment effect.

**Table 1:** Characteristics of the simulated populations

Variable(s)	Scenario	Setting(s)	Description(s)
$q$	Scenario 1	c(1.5,1.5,1.5,1.5,1.5)	Parameter in the survival function. If a subject suffers more events than specified distributions, the last parameter specified here is used to generate times corresponding to later events.
	Scenario 2		
	Scenario 3		
	Scenario 4		
${\beta }_{0}$	Scenario 1	c(6,5.5,5,4,3)	
	Scenario 2		
	Scenario 3		
	Scenario 4		
$T$	Scenario 1	$T\sim Bernoulli (0.5)$	Binary measured treatment indicator.
	Scenario 2		
	Scenario 3		
	Scenario 4		
${X}_{cov}$	Scenario 1	${X}_{1}\sim {X}_{5} \sim Normal (\text{0,1})$	Five independent covariates with standardized normal distribution. They cause collider bias in the PWP models.
	Scenario 2		
	Scenario 3		
	Scenario 4		
$HR$	Scenario 1	0.75	Hazard ratio of the treatment.
	Scenario 2		
	Scenario 3	1	
	Scenario 4		
${HR}_{cov}$	Scenario 1	0.9	Hazard ratio of the five independent covariates. They assess the magnitude of collider bias, the larger effect size corresponds to the larger collider bias.
	Scenario 2	1.2	
	Scenario 3	0.9	
	Scenario 4	1.2	

For each scenario, the follow-up time was set as two years. Censor and risk-free interval were not under consideration. Sample sizes of 100, 300, and 500 were simulated, with 10000 simulations for each scenario.

### Methods for data analysis

Each simulated dataset was analyzed using the following methods: (1) Cox model for time-to-first-event [11], (2) AG model [12], (3) Lin-Wei-Yang-Ying model (LWYY model) [37], (4) Poisson regression model [5], (5) negative binomial model (NB model) [6], (6) PWP model [13], (7) PWP model with robust variance [38], and (8) weighted PWP model. Two weighted PWP models were considered: the fully weighted model included all five covariates for entropy balance, whereas the partly weighted PWP model included only three covariates for entropy balance. Besides, to ensure comparability across these models, we evaluated the performance of covariate adjustment via outcome regression for the first seven models, using the same covariate information as the weighted PWP model: fully outcome regression models and partly outcome regression models.

We also performed some supplementary analyses. First, we used inverse probability weighting to replace entropy balance in the weighting phase, with stabilized weights calculated as ${sw}_{ki}=\frac{P({A}_{i})}{P({A}_{i}|{X}_{cov, i})}$ in the $k$th risk set for individual $i$ [39, 40]. Here, $P({A}_{i}|{X}_{cov, i})$ is the estimated propensity score, and $P({A}_{i})$ is the estimated marginal probability of having the treatment for participant $i$. The numerator is the probability of the observed treatment level (i.e. the observed frequency). The denominator is the conditional probability of the observed treatment level given the observed covariates, estimated by logistic regression. Second, in practice, the risk set may become extremely small for later strata, making covariate balancing by weighting less ideal [1, 23]. We truncated the dataset and used standardized mean difference (SMD) to assess covariate balance after weighting [41]. The number of recurrent events $K$ included in the final analysis was restricted to $1\le K<{k}_{max}$, where ${k}_{max}$ is the first stratum that could not satisfy SMD < 0.1 for any covariate under study.

### Performance measures

Bias, defined as the difference between the estimated value and the true parameter value, was used to quantify whether the estimate targeted the true value of interest [34]. The null hypothesis that the treatment has no effect was rejected if the two-sided 95% confidence interval (CI) for the treatment effect estimate did not include 1. Under the scenario where the null hypothesis was true, the type I error rate ${\widehat{p}}_{T1E}$ was calculated as the number of simulations with null hypothesis rejection divided by the total number of simulations. Its Monte Carlo standard error can be calculated by $\sqrt{\frac{{\widehat{p}}_{T1E}(1-{\widehat{p}}_{T1E})}{{n}_{sim}}}$ and compared to the two-sided nominal significance level of 0.05, where ${n}_{sim}$ is the total number of simulations. For models controlling the type I error rate at 0.05, we further compared their statistical power under the scenarios where the null hypothesis was not true, defined as the number of simulations with null hypothesis rejection divided by the total number of simulations.

### Simulation results

**Fig. 3:**
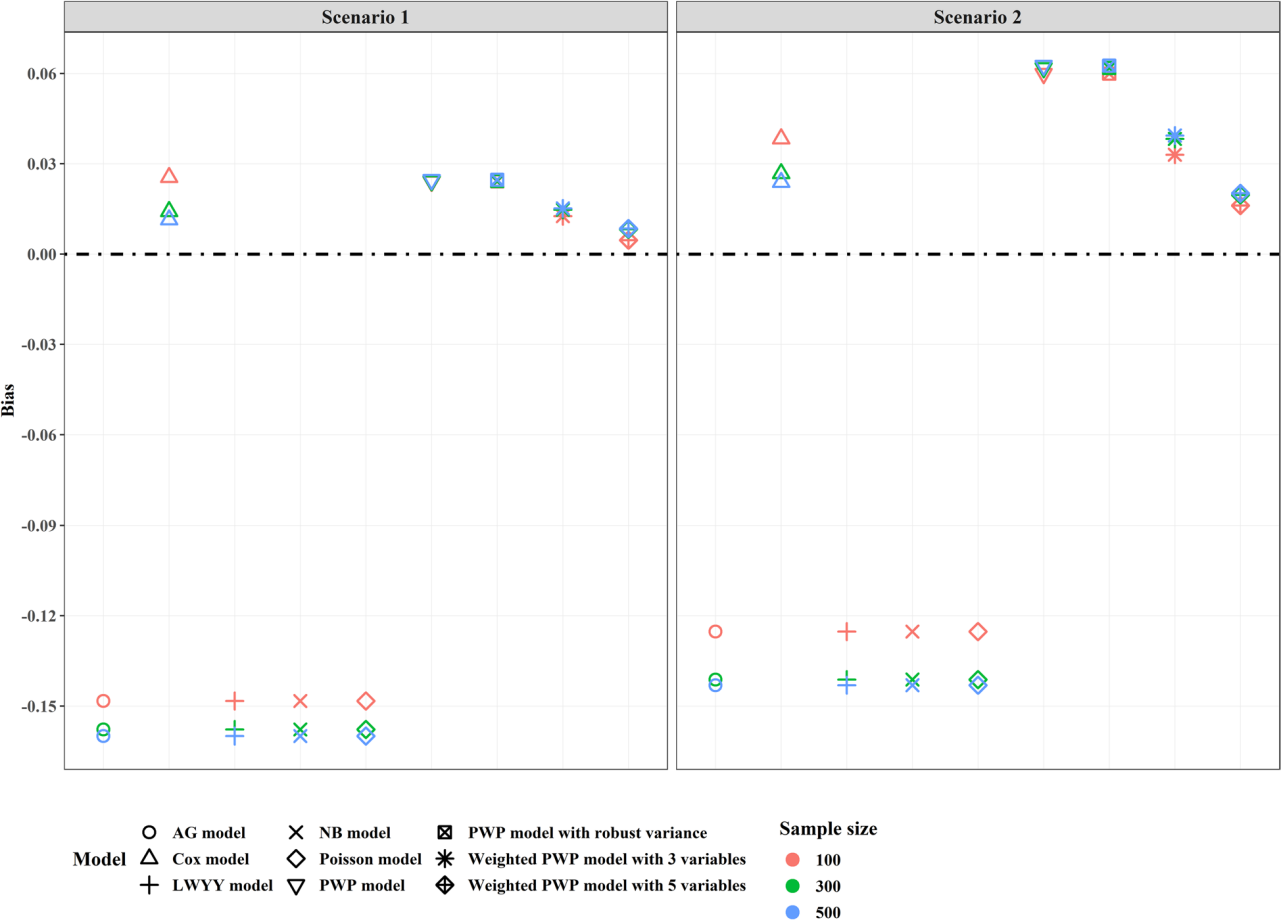
Bias for the estimated treatment effect for different models under varying sample sizes and covariate effect sizes

Based on 10000 simulations for each scenario, we found that the weighted PWP model had the smallest bias, especially when all covariates were included (Fig. 3). In our simulations, all covariates were measured and remained constant over time, resulting in consistent point estimates from the AG, LWYY, Poisson, and NB models. However, these four models consistently overestimated treatment effects. As anticipated, the PWP models tended to underestimate effect sizes in all scenarios, particularly when covariate effect sizes were substantial. The results from the Cox model were unstable, often producing biased estimates, especially when sample sizes were small.

Table 2 shows type I error rates for different models under varying sample sizes and covariate effect sizes, with bold text presenting inflated type I error rates. We found that the AG and Poisson models led to extremely inflated type I error rates (more than 0.50). However, LWYY and NB models were able to control type I error rates in most scenarios. This is because the LWYY model uses the robust sandwich estimate of the covariance matrix, and the score residuals used in computing the middle part of the sandwich estimate are aggregated over identical individuals [42]. The NB model assumes conditional independence between events given a gamma-distributed random effect [9]. Both models are partially correct for the dependency structure, resulting in more robust variance estimates. Consistent with findings by Wei and colleagues [22], we found that the PWP model, including the version with robust variances, frequently rejected the null hypothesis of no treatment effect, especially when covariates effect sizes were substantial.

**Table 2:** Type I error rates for different models under varying sample sizes and effect sizes for covariates

Scenario	Sample size	Type I error rates								
		Cox model	AG model	LWYY model	NB model	Poisson model	PWP model	PWP model with robust variances	Weighted PWP model with 3 variables	Weighted PWP model with 5 variables
Scenario 3	100	0.0502	**0.5502**	**0.0546**	0.0415	**0.5502**	**0.0963**	**0.0685**	0.0517	0.0413
	300	0.0522	**0.5611**	0.0523	0.0382	**0.5611**	**0.1004**	**0.0591**	0.037	0.0196
	500	0.0527	**0.5561**	0.0527	0.0371	**0.5561**	**0.1058**	**0.0575**	0.0323	0.0192
Scenario 4	100	0.0531	**0.6072**	**0.0586**	0.0446	**0.6072**	**0.1777**	**0.0796**	0.0413	0.0181
	300	0.0497	**0.6079**	0.053	0.0407	**0.6079**	**0.1848**	**0.0624**	0.028	0.0035
	500	0.0522	**0.6051**	0.0524	0.0389	**0.6051**	**0.1851**	**0.0583**	0.0234	0.0043

The bold T1E rates are those with a lower limit of the two-sided 95% CI above the significance level of 0.05

**Fig. 4:**
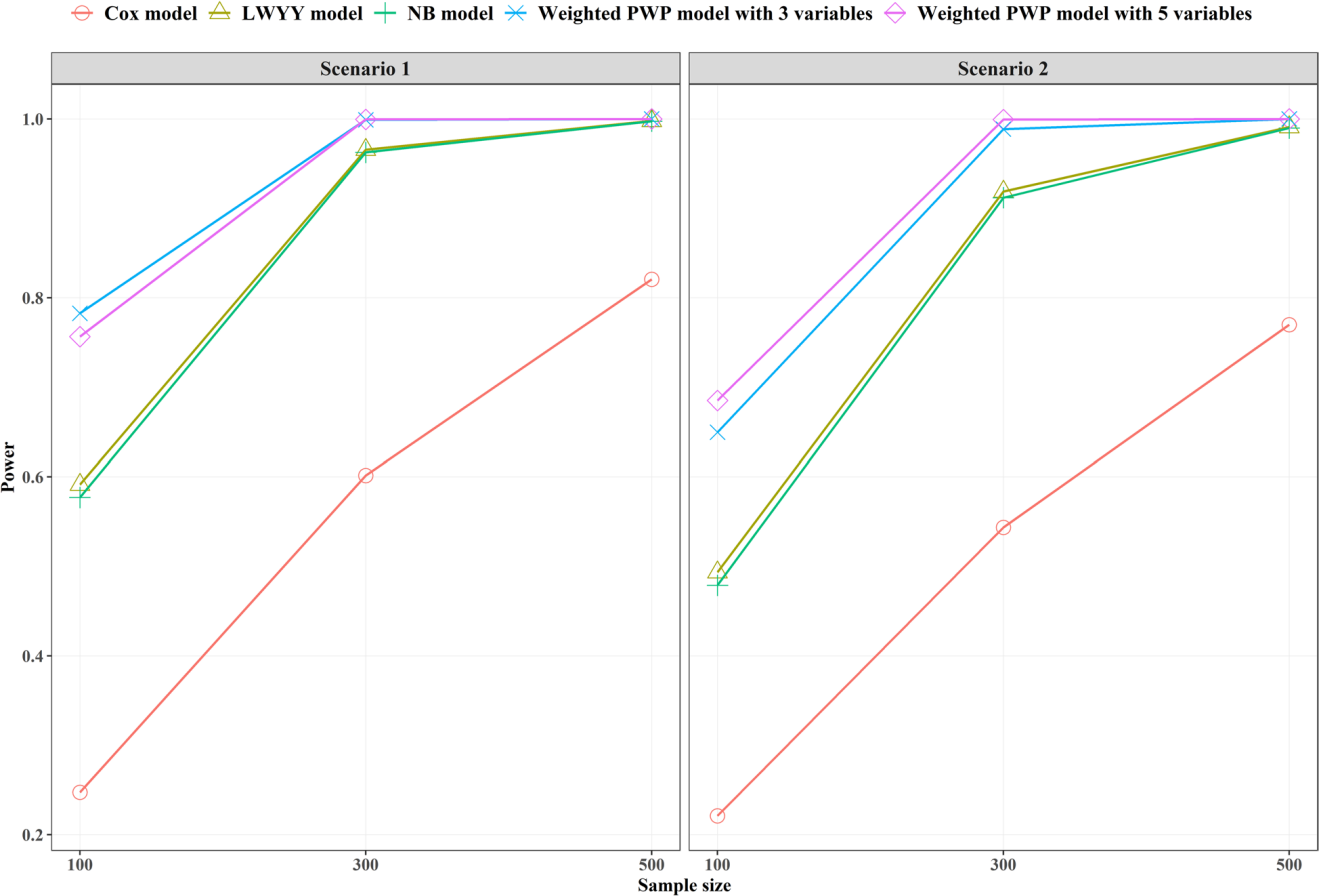
Statistical power for models without inflated type I error rate under varying sample sizes and covariate effect sizes

We also assessed the statistical power of alternative models that did not exhibit inflated type I error rates (Fig. 4). The trend of power estimates was consistent across these models, increasing with larger sample sizes and decreasing covariate effect sizes. Among the models, the weighted PWP models consistently demonstrated the highest power in all simulation settings. With sufficiently large sample sizes, the extent of covariate information collected had minimal influence on the statistical power of the weighted PWP models. The Cox model showed the lowest statistical power because it utilizes the least relevant information among all methods. The LWYY model utilizes time-to-event information more directly, resulting in a slightly higher statistical power compared to the NB model, particularly when the sample size is small and the covariate effect is strong. However, the difference may not be pronounced, likely due to the random effects term in the NB model capturing partial information.

Table S1 (Supplementary Material 1) presents the bias and type I error rates for covariate adjustment via outcome regression. When multivariate regression models were implemented, we observed some inflation in the type I error rates across all models. As noted in previous studies, adjusting for covariates in a regression model can increase the probability of false positives when dealing with small sample sizes and time-to-event outcomes [43, 44]. This inflation occurs because outcome regression model may lead to overstratification in such contexts, where the number of covariates is too high relative to the number of observed events [45]. Consequently, it is crucial to consider both the overall sample size and expected event rate when determining how many covariates to include in the analysis. However, in practical situations, numerous influencing factors need to be considered. This underscores the importance of using a weighted approach for balancing covariates as a more rational strategy. The PWP models, which also incorporate time-to-event outcomes, are not immune to type I error rate inflation, despite their seemingly lower bias. However, the reduced bias may be contingent upon correct model specification, which can be challenging to achieve in practice.

Table S2 (Supplementary Material 1) shows the bias for different weighting and truncation strategies. We found that estimates became unstable when the number of recurrent events was unrestricted and stabilized weights obtained by logistic regression were used. This instability arises because the risk set becomes very small in later strata, making inverse probability weights unreliable. Using entropy balance facilitates the attainment of more robust effect estimates. When we restricted the number of recurrent events, all weighting strategies yielded stable and nearly consistent results. These indicate that the information from later strata with mall risk sets may have limited utility. Therefore, the entropy balance approach could serve as a more effective weighting strategy.

### Real data application: bladder cancer recurrences

We applied the methods to a real dataset from “The Veterans Administration Study of Chemoprophylaxis for Recurrent Stage I Bladder Tumors”. This dataset, renowned for its recurrent event data, has been frequently used in previous studies to illustrate and validate related methodologies, including the PWP models [1, 21, 46]. The primary objective of the treatment in this dataset was to prevent the recurrence of bladder cancer following the excision of superficial bladder tumors. A total of 38 patients were randomized to receive Thiotepa, while 47 patients were assigned to a placebo group. Two baseline variables were collected: the number of initial tumors and the size of the largest initial tumor [22]. Subjects were followed for up to 64 months, and the event of interest was tumor recurrence. We used all eight models to estimate the effect of Thiotepa.

**Fig. 5:**
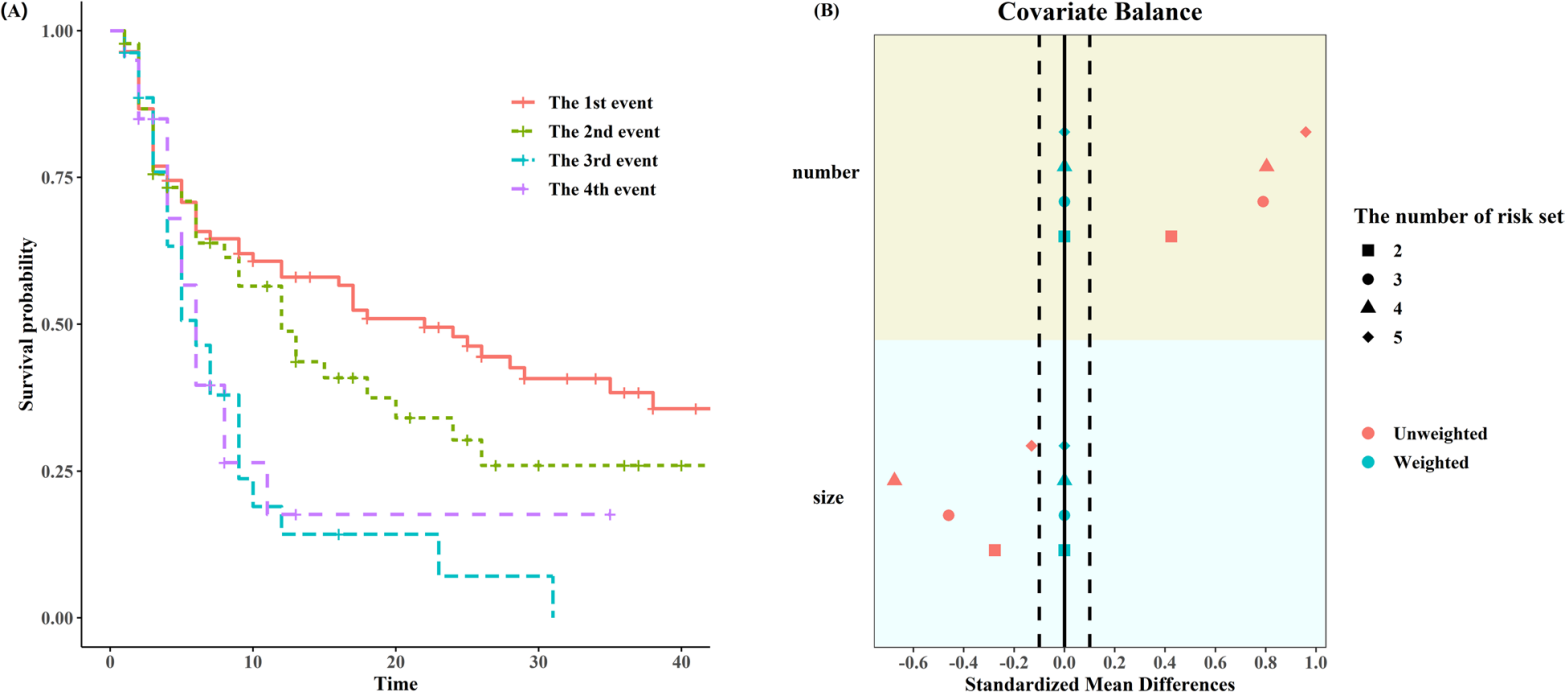
Characteristics of the Bladder Cancer Recurrences data. **A** Survival curve for the first four tumor recurrences; (**B**) Covariate balance before and after weighting in different risk sets

Approximately 55% (47/85) of patients experienced at least one recurrence, resulting in a total of 132 recurrences. In the placebo group, mean number of recurrences was 1.85, with a range from 0 to 9. Mean number of recurrences was 1.18 in the Thiotepa group, ranging from 0 to 7. Figure. 5A shows the survival curve for the first four tumor recurrences, we found that survival probability decreased with the increase of the number of recurrences. Even all patients were randomized to two groups before treatment, covariates become imbalanced in the risk set for subsequent recurrent events and the degree of imbalance seems to be on the rise. After weighting, the covariates achieve a state of balance (Fig. 5B).

Table 3 shows the results of eight analytical approaches used to estimate the treatment effect. The Cox model fit time to the first event, thereby excluding 64.39% (85/132) of the subsequent recurrences. Both the AG and LWYY models produce identical point estimates, as there are no covariates that varied over time in relation to the event history. However, a notable distinction between the AG and LWYY models lies in their confidence intervals, which result from their unique methodologies for estimating the variability of these estimates. Consistent with the simulation findings, the AG, LWYY, Poisson, and NB models consistently estimated higher effects than the PWP models. In contrast, the weighted PWP model provided estimates that were intermediate between these models. The estimates from the AG and Poisson models were statistically significant, possibly due to type I error inflation.

**Table 3:** Results of eight analytical approaches for treatment effect: effects of treatment on tumor recurrences in bladder cancer patients

Model	Effects	95% CI
Cox model	0.6958	(0.3844, 1.259)
AG model	0.6696	(0.4669, 0.9603)
LWYY model	0.6696	(0.3808, 1.177)
Poisson model	0.6681	(0.4662, 0.9575)
NB model	0.7425	(0.4172, 1.3214)
PWP model	0.8893	(0.6118, 1.293)
PWP model with robust variance	0.8893	(0.6062, 1.305)
Weighted PWP model	0.8425	(0.511, 1.389)

## Discussion

In this study, we proposed a novel alternative approach, the weighted PWP model, which is easy to implement and easy to understand for modelling recurrent event data, while other common methods for analyzing recurrence data had inflated type I error rates and biased estimates when the baseline hazard function is different for each recurrence. The weighted PWP model has been validated with small bias, well-controlled type I error rate, and high statistical power in simulated RCTs. In addition, we applied this model to evaluate the efficacy of Thiotepa on the recurrence of bladder cancer following the excision of superficial bladder tumors.

Interpretation of treatment effects derived from weighted PWP models requires careful consideration. When the effect size is interpreted as a hazard ratio, it represents the ratio of instantaneous hazards. However, this lacks causal interpretability due to the inherent built-in selection bias, as it involves comparison between two groups of individuals who are not comparable—particularly when examining period-specific hazard ratios [47]. In contrast, when the effect size is understood as a cumulative hazard ratio, it becomes causally interpretable and numerically equivalent to the hazard ratio, provided that all relevant assumptions are met [24, 25]. Therefore, if these assumptions hold, the effect size may be referred to as the hazard ratio; however, when seeking a causal explanation, it is more appropriate to designate it as the cumulative hazard ratio.

Given the natural progression of diseases, many conditions demonstrate an increased risk of recurrence as the frequency of previous episodes rises. For example, some studies indicated that a prior fall elevated the risk of subsequent falls by threefold [48] and a progressive shortening of the interval length between hospitalization episodes was observed in patients with heart failure [15]. In these scenarios, the PWP model demonstrates a superior ability to characterize the disease progression by allowing for varying baseline hazard function, while other common methods for analyzing recurrence data had inflated type I error rates and biased estimates. However, it is essential to exercise caution regarding the application of the traditional PWP model in RCTs. It constrains the risk set to only those who have experienced previous events, potentially compromising the initial randomization and introducing collider bias, which often goes unnoticed by many researchers [28]. Besides, covariate adjustment via outcome regression is often considered in RCTs to increase power and guard against chance imbalances [49–52]. However, our findings suggest that this practice may lead to inflated type I error rates in the PWP models, especially when the sample size is small.

To our knowledge, no study has attempted to weight individuals in each risk set to reconstruct balance between groups and evaluate the statistical performance of this practice. The proposed weighted PWP model effectively addressed the underestimation of effect size observed in the traditional PWP model, even though only a subset of covariates were collected. Moreover, it well controlled type I error rates and demonstrated higher statistical power compared to other models. As hazard ratio is a relative measure, whose explanation is not intuitive, and researchers often use annualized rate as the summary measure in estimand. We agree with Hengelbrock et al. [53] that it is recommendable to report both the estimates of the mean frequency number of events and the estimates of the weighted PWP model, choosing one as a supplementary analysis according to the fitness of the data characteristics to the model assumptions.

We recommend using entropy balance to obtain weights due to its flexibility. Common logistic regression-derived inverse probability weights depend heavily on correct model specification [54], and extreme estimates can arise with a small sample size [40], a challenge that is particularly unavoidable in the PWP model due to the progressively decreasing risk set sizes. Truncating the dataset when the risk set is too small can help achieve covariate balance. This point could also be considered when determining follow-up duration.

### Limitations

First, we only focused on the most common situation in which the baseline hazard increased with the number of events. And because of the technical gap in generating data based on calendar time when baseline hazards vary, we only simulated gap time scales. However, according to the same principle, our conclusion should hold under other scenarios. Second, while we attempted the most prevalent robust weights and the most efficient entropy balance approach, other weighting methods may warrant exploration. As long as the covariates are balanced, the impact of different weights is expected to be modest. Third, unmeasured heterogeneity was not addressed in our study. A previous study has demonstrated that the PWP frailty model can reduce bias; however, its effectiveness relies on the correct specification of the distribution and parameters of random subject effect [53], which may be challenging to achieve in practice. Therefore, further research is necessary to address unmeasured heterogeneity. Our study indicated that when employing PWP models, even in RCTs, it is essential to collect influencing factors as comprehensively as possible. Fourth, our simulation study focused on constant treatment effects and did not account for event-specific treatment effects. Theoretically, the weighting method proposed in this study can deal with collider bias that the traditional PWP models may introduce under both settings. The conclusions drawn from this study remain unaffected. Fifth, we commonly determine whether baseline hazard changes with the number of recurrent events, based on the understanding of disease characteristics. There is a lack of specific methods to test this assumption, which warrants further studies. Finally, the applicability of this method may be constrained by the assumptions outlined in the Method section. Therefore, it warrants further studies to investigate potential avenues for relaxing these assumptions.

## Conclusions

To conclude, we have attempted to identify statistical models that best estimate intervention effects when the baseline hazard function differs for each recurrence in RCTs. We developed a weighted PWP method and compared it with existing common methods by evaluating bias, type I error rates, and statistical power across different sample sizes, covariate effect sizes, and true intervention effects. The simulation results indicated that the proposed weighted PWP model showed the best performance. Other models, including the Cox model on time-to-first-event, Poisson, NB, AG, LWYY, and PWP models, all showed different degrees of bias and inflated type I error rates.

## Supplementary Information

Supplementary Material 1.

## Data Availability

The data that support the findings of this study are openly available in R package survival at https://CRAN.R-project.org/package=survival.

## Abbreviations

RCTs: Randomized controlled trials
PWP: Prentice, Williams, and Peterson
NB: Negative binomial
AG: Andersen-Gill
LWYY: Lin-Wei-Yang-Ying
PWP-CP: PWP counting process
PWP-GT: PWP gap time
HR: Hazard ratio
T1E: Type I error
SMD: Standardized mean difference
CI: Confidence interval
