# Supplementary material for: A new method for dealing with collider bias in the PWP model for recurrent events in randomized controlled trials
Source: BMC Med Res Methodol. 2025 May 26;25:142. doi: 10.1186/s12874-025-02596-0 (PMC12105184; doi:10.1186/s12874-025-02596-0)
Supplement: Supplementary file 1 — Supplementary Material 1. [file 12874_2025_2596_MOESM1_ESM.docx]

**Supplemental Materials for “A new method for dealing with collider bias in the PWP model for recurrent events in randomized controlled trials”**

**Method S1.** Identication proofs.

**Table S1.** Bias and type I error rates for the outcome regression models under varying sample sizes and effect sizes for covariates.

**Table S2.** Bias for different weighting and truncation strategies under varying sample sizes and effect sizes for covariates.

**Method S1. Identication proofs.**

We provide proofs for the $\frac{H_{A=1}\left( t \right)}{H_{A=0}\left( t \right)}$ identification formula under assumptions, mainly about $h_{A=a,k}\left( t \right)$ can be identified by $h_{k}\left( t|A=a \right)$.

First, according to the definition of average cumulative hazard ratio, we can get

$\frac{H_{A=1}\left( t \right)}{H_{A=0}\left( t \right)}=\frac{\sum_{k=1}^{K} \varphi_{k}H_{A=1,k}\left( t \right)}{\sum_{k=1}^{K} \varphi_{k}H_{A=0,k}\left( t \right)}=\frac{\sum_{k=1}^{K} \varphi_{k}\int_{0}^{t} h_{A=1,k}\left( u \right)du}{\sum_{k=1}^{K} \varphi_{k}\int_{0}^{t} h_{A=0,k}\left( u \right)du}$.

Next, we show that we can identify $h_{A=a,k}\left( t \right)$ as $h_{k}\left( t|A=a \right)$. For simplicity, we omit the subscript $k$. Thus, $h_{A=a,k}\left( t \right)$ is denoted as $h_{A=a}\left( t \right)$ and $h_{k}\left( t|A=a \right)$ is denoted as $h\left( t|A=a \right)$ in the proof below. We intend to identify $h_{A=a}\left( t \right)$ as $h\left( t|A=a \right)=\frac{E[dN\left( t | a \right)]}{E[\tilde{Y}(t|a)]}$. Let $T^{A=a,C=\infty}$ denote the potential event time with treatment group $a$, $a=0, 1$, and there was no censoring, namely that the censoring time was $C=\infty$. Let $C^{A=a}$ denote the potential censoring time under treatment level $A=a$. Let $O^{A=a}=min\{T^{A=a,C=\infty},C^{A=a}\}$ and $\delta^{A=a}=I\{T^{A=a,C=\infty}\leq C^{A=a}\}$ represent the observed time and the event indicator, respectively, had we intervened and set $A=a$. We denote the potential outcomes’ event counting processes by $N^{A=a}\left( t \right)=\delta^{A=a}I\{O^{A=a}\leq t\}$. We can obtain the risk indicator $\tilde{Y}(t) = I\{O\geq t\}$. The indicator of the jump in the process $N(t)$ during the interval $[t, t+dt)$, is denoted by $dN(t)$. Let $N\left( t | A=a \right)=N\left( t \right)I\{A=a\}$, $dN\left( t | A=a \right)=dN\left( t \right)I\{A=a\}$, and $\tilde{Y}\left( t | A=a \right)=\tilde{Y}(t)I\{A=a\}$ be the analogous quantities in each treatment.

The assumption 2) censoring is non-informative can be written as (Axelrod and Nevo 2023)

$\Pr\left( t\leq O^{A=a}<t+dt,\delta^{A=a}=1 | O^{A=a}\geq t \right)=\Pr\left( t\leq O^{A=a,C=\infty}<t+dt | T^{A=a,C=\infty}\geq t \right)$.

Therefore, $h_{A=a}\left( t \right)$ can be writen as

$$h_{A=a}\left( t \right)=\lim_{dt\to0} {(dt)}^{-1}\Pr\left( t\leq O^{A=a,C=\infty}<t+dt | T^{A=a,C=\infty}\geq t \right)$$

$$=\lim_{dt\to0} {(dt)}^{-1}\Pr\left( t\leq O^{A=a}<t+dt,\delta^{A=a}=1 | O^{A=a}\geq t \right)$$

$$=\frac{\lim_{dt\to0} \left( dt \right)^{-1}\Pr\left( t\leq O^{A=a}<t+dt,\delta^{A=a}=1 \right)}{\Pr\left( O^{A=a}\geq t \right)}$$

$=\frac{\Pr[dN^{A=a}\left( t \right)=1]}{\Pr(O^{A=a}\geq t)}$.

For the numerator $\Pr\left[ dN^{A=a}\left( t \right)=1 \right]$, under assumption 1) SUTVA and 4) randomization we have

$\Pr\left[ dN^{A=a}\left( t \right)=1 \right]=\Pr\left[ dN\left( t \right)=1 | A=a \right]=\frac{\Pr[dN\left( t \right)=1,A=a]}{Pr(A=a)}=\frac{E[dN\left( t | A=a \right)]}{Pr(A=a)}$.

Similarly, for the denominator $\Pr(O^{A=a}\geq t)$, we have

$\Pr(O^{A=a}\geq t)=\Pr\left( O\geq t | A=a \right)=\frac{Pr(O\geq t,A=a)}{Pr(A=a)}=\frac{E[\tilde{Y}(t|A=a)]}{Pr(A=a)}$.

In total, we get $h_{A=a}\left( t \right)=\frac{{E[dN\left( t | A=a \right)]}/{Pr(A=a)}}{{E[\tilde{Y}(t|A=a)]}/{Pr(A=a)}}=\frac{E[dN\left( t | A=a \right)]}{E[\tilde{Y}(t|A=a)]}=h\left( t|A=a \right)$.

Then, we have

$\frac{H_{A=1}\left( t \right)}{H_{A=0}\left( t \right)}=\frac{\sum_{k=1}^{K} \varphi_{k}H_{A=1,k}\left( t \right)}{\sum_{k=1}^{K} \varphi_{k}H_{A=0,k}\left( t \right)}=\frac{\sum_{k=1}^{K} \varphi_{k}\int_{0}^{t} h_{A=1,k}\left( u \right)du}{\sum_{k=1}^{K} \varphi_{k}\int_{0}^{t} h_{A=0,k}\left( u \right)du}=\frac{\sum_{k=1}^{K} \varphi_{k}\int_{0}^{t} h_{0k}\left( u \right)e^{\beta}du}{\sum_{k=1}^{K} \varphi_{k}\int_{0}^{t} h_{0k}\left( u \right)du}=e^{\beta}$.

**Reference:**

Axelrod R, Nevo D (2023) A sensitivity analysis approach for the causal hazard ratio in randomized and observational studies. Biometrics 79:2743–2756. https://doi.org/10.1111/biom.13797

**Table S1.** Bias and type I error rates for the outcome regression models under varying sample sizes and effect sizes for covariates.

|  | Effect sizes for covariates | Sample size | Cox model | | AG model | | LWYY model | | NB model | | Poisson model | | PWP model | | PWP model with robust variance | |
| --- | --- | --- | --- | --- | --- | --- | --- | --- | --- | --- | --- | --- | --- | --- | --- | --- |
|  |  |  | Bias | T1E | Bias | T1E | Bias | T1E | Bias | T1E | Bias | T1E | Bias | T1E | Bias | T1E |
| Partly outcome regression model with 3 covariates | 0.9 | 100 | 0.0183 | **0.0563** | -0.1485 | **0.5252** | -0.1485 | **0.0579** | -0.1543 | 0.0513 | -0.1485 | **0.5252** | 0.0087 | **0.0702** | 0.0087 | **0.0753** |
|  |  | 300 | 0.0078 | **0.0545** | -0.1584 | **0.5334** | -0.1584 | 0.053 | -0.1618 | 0.0436 | -0.1584 | **0.5334** | 0.0094 | **0.075** | 0.0094 | **0.0595** |
|  |  | 500 | 0.0058 | 0.0531 | -0.1603 | **0.5367** | -0.1603 | 0.049 | -0.1631 | 0.0424 | -0.1603 | **0.5367** | 0.0100 | **0.0765** | 0.0100 | **0.0570** |
|  | 1.2 | 100 | 0.0226 | **0.0584** | -0.1309 | **0.5573** | -0.1309 | **0.0610** | -0.1405 | **0.0580** | -0.1309 | **0.5573** | 0.0247 | **0.1284** | 0.0247 | **0.0867** |
|  |  | 300 | 0.0139 | 0.0530 | -0.1425 | **0.5639** | -0.1425 | **0.0554** | -0.1514 | 0.0476 | -0.1425 | **0.5639** | 0.0280 | **0.1346** | 0.0280 | **0.0649** |
|  |  | 500 | 0.0114 | 0.0499 | -0.1438 | **0.556** | -0.1438 | **0.0558** | -0.1524 | 0.0465 | -0.1438 | **0.556** | 0.0288 | **0.1363** | 0.0288 | **0.0589** |
| Fully outcome regression model with 5 covariates | 0.9 | 100 | 0.0130 | **0.0597** | -0.1497 | **0.5147** | -0.1497 | **0.0602** | -0.1577 | **0.0600** | -0.1497 | **0.5148** | -0.0027 | **0.0554** | -0.0027 | **0.0776** |
|  |  | 300 | 0.0034 | **0.0574** | -0.1587 | **0.5188** | -0.1587 | 0.0540 | -0.1643 | 0.0455 | -0.1587 | **0.5188** | -0.0015 | 0.0473 | -0.0015 | **0.0565** |
|  |  | 500 | 0.0018 | **0.0545** | -0.1605 | **0.5234** | -0.1605 | 0.0515 | -0.1652 | 0.0434 | -0.1605 | **0.5234** | -0.0010 | 0.0496 | -0.0010 | **0.0565** |
|  | 1.2 | 100 | 0.0114 | **0.0636** | -0.1352 | **0.5158** | -0.1352 | **0.0661** | -0.1465 | **0.0659** | -0.1352 | **0.5158** | -0.0037 | **0.0551** | -0.0037 | **0.0857** |
|  |  | 300 | 0.0040 | **0.0554** | -0.1442 | **0.5121** | -0.1442 | **0.0559** | -0.1556 | 0.0477 | -0.1442 | **0.5121** | -0.0018 | 0.0516 | -0.0018 | **0.0637** |
|  |  | 500 | 0.0020 | 0.0536 | -0.1447 | **0.5164** | -0.1447 | 0.0537 | -0.1561 | 0.0480 | -0.1447 | **0.5164** | -0.0011 | 0.0516 | -0.0011 | **0.0579** |

Note:

Bias was evaluated in the context where the effect size for treatment was $HR=0.75$.

T1E was evaluated in the context where the effect size for treatment was $HR=1$.

The bold T1E rates are those with a lower limit of the two-sided 95% CI above the significance level of 0.05.

**Table S2.** Bias for different weighting and truncation strategies under varying sample sizes and effect sizes for covariates.

|  | Effect sizes for covariates | Sample size | Stabilized weights | | Entropy balance | |
| --- | --- | --- | --- | --- | --- | --- |
|  |  |  | Untruncated | Truncated | Untruncated | Truncated |
| With 3 covariates | 0.9 | 100 | 6590.3940 | 0.0114 | 0.0126 | 0.0126 |
|  |  | 300 | 4471.3631 | 0.0146 | 0.0147 | 0.0147 |
|  |  | 500 | 0.0157 | 0.0152 | 0.0152 | 0.0152 |
|  | 1.2 | 100 | 8684.2690 | 0.0311 | 0.0330 | 0.0329 |
|  |  | 300 | 0.0407 | 0.0376 | 0.0382 | 0.0383 |
|  |  | 500 | 4034.7120 | 0.0390 | 0.0394 | 0.0394 |
| With 5 covariates | 0.9 | 100 | 43288.8584 | 0.0069 | 0.0046 | 0.0046 |
|  |  | 300 | 39344.1751 | 0.0082 | 0.0082 | 0.0082 |
|  |  | 500 | 40228.6078 | 0.0087 | 0.0085 | 0.0085 |
|  | 1.2 | 100 | 85982.5696 | 0.0180 | 0.0161 | 0.0161 |
|  |  | 300 | 30826.6514 | 0.0203 | 0.0197 | 0.0197 |
|  |  | 500 | 73841.5916 | 0.0211 | 0.0202 | 0.0202 |
